# KaRhab: an international online registry for cardiac rhabdomyomas

**DOI:** 10.1186/s13023-024-03470-w

**Published:** 2025-01-30

**Authors:** Vera-Maria Herrmann, Maria Arelin, Caroline G. Bergner, Julia Herrmann, Paula Janz, Henriette Kiep, Annika Mueller, Steffen Syrbe, Robert Wagner, Bardo Wannenmacher, Nadine Wolf, Michael Weidenbach, Vincent Strehlow

**Affiliations:** 1 Institute of Human Genetics, Leipzig University Medical Center, Leipzig, Germany; 2 Department of Neuropediatrics, University Hospital for Children and Adolescents, Leipzig, Germany; 3 Department of Neurology, Leipzig University Medical Center, Leipzig, Germany; 4 Department of Pediatric Cardiology, Heart Center Leipzig - University Hospital, Leipzig, Germany; 5 Department of Neonatology, Leipzig University Medical Center, Leipzig, Germany; 6 Department of Pediatrics, University Hospital Halle, Halle, Germany; 7 Division of Paediatric Epileptology, Centre for Paediatrics and Adolescent Medicine, University Hospital Heidelberg, Heidelberg, Germany; 8 Medical Office Dr. Robert Wagner, Leipzig, Germany

**Keywords:** Cardiac rhabdomyoma, Tuberous sclerosis complex, mTOR inhibitors, Patient registry

## Abstract

**Background:**

Cardiac rhabdomyoma (RHM) is considered one of the most frequent benign heart tumors in children. However, encounters with cardiac RHM in clinical practice remain rare. Clinical information is primarily available in the form of single case reports or smaller studies with a shortage of large-scale reviews encompassing a substantial number of cases.

**Results:**

In order to congregate existing and future information on cardiac RHM we established a web-based cardiac RHM online registry using an online survey tool. In addition we integrated previously published data from individual case reports and case series. The evaluation of this paper is intended to provide a brief overview of the cohort that we have been able to include so far. Our findings mainly confirm the previous knowledge on cardiac RHM. At the same time, our cohort shows a clear heterogeneity in the treatment methods with regard to rhabdomyomas requiring therapy and revealed a bias between literature data and our registry data with regard to symptoms and need for therapy.

**Conclusion:**

In the view of the heterogeneity of treatment methods, a systematic overview of cardiac RHM is all the more important, especially as specific drug treatment options now exist. The registry should not just provide a comprehensive and informative overview of causes, time course, symptoms and therapeutic options of cardiac RHM but also facilitate information sharing among clinicians and researchers and serve as a basis for future clinical and pharmacological studies.

**Supplementary Information:**

The online version contains supplementary material available at 10.1186/s13023-024-03470-w.

## Background

Among cardiac tumors in childhood and during fetal period, cardiac RHM accounts for the largest proportion of approximately 40–60% of cases [1, 2]. Nevertheless, the encounter with primary cardiac tumors in the pediatric context is rare overall. While post-mortem examinations show a prevalence of 0.0017 to 0.28 for primary cardiac tumors [2], the frequency of antenatally diagnosed cardiac tumors is 0.14% [3]. Therefore, a certain rate of undiagnosed cases is possible. Histologically, cardiac RHM is characterized by particular pathognomic hallmarks, spider cells, arising from embryonic myocytes [4]. It typically affects the ventricular myocardium but can also be found in other cardiac structures such as the right or left atrium or the valves [5]. In echocardiography, it presents as a hyperechogenic mass that contrasts with the rest of the myocardium and can already be detected in prenatal sonography, typically after the 20th week of gestation [6, 7]. Cardiac RHM vary in number, size and location [8]. Depending on these features symptoms range from asymptomatic to severe cardiac complications including obstruction of blood flow, arrhythmia, heart failure and fetal or neonatal death [8, 9]. Cardiac RHM tend to regress spontaneously after birth in the majority of cases [1, 5]. In the occurrence of the severe complications described above, various therapeutic options are available. These include catheter interventions for duct dependent situations [10], surgical interventions for obstruction and/or more complex anatomical conditions [9] and pharmacological treatments. Among these anticongestive therapy [11], prostaglandins [12] and antiarrhythmic drugs [13] were the most prevalent in the past. A vast majority of cases with cardiac RHM is associated with Tuberous Sclerosis Complex (TSC) [6], a rare genetic disorder characterized by neurological findings, characteristic skin lesions and various tumor types in different organ systems. It is caused by pathogenic variants in the genes *TSC1* or *TSC2* that are involved in the mTOR signaling pathway. The growing understanding of the underlying pathophysiological mechanisms has recently led to the investigation of substances interfering with the mammalian target of rapamycin (mTOR) pathway, e.g. Sirolimus, Everolimus, in patients with TSC [14]. Single trial reports in patients with cardiac RHM show promising results but so far results of large-scaled studies are pending [15, 16]. Dosing, duration of therapy and long-term outcome are questions that still need to be answered. In the view of increasingly improving diagnostic methods and expanding therapeutic options there is a growing need to obtain a systematized overview of the clinical and genetic characteristics, symptomatology and therapies in patients with cardiac rhabdomyomas. Due to its low incidence, recruitment of cohorts with high case numbers of cardiac RHMs is challenging, even in specialized centers. For this reason, we established an online registry aiming not only for medical professionals, but also particularly for affected individuals, their parents and relatives. To minimize language and access barriers, the questionnaire is provided in multiple languages as to make use and response as easy as possible. Currently the questionnaire is available in German, English, French and Italian but we are preparing the translation into other languages. Beyond that, the resulting registry shall serve as a basis for further extensive research, e.g. genotype-phenotype relations, clinical and therapeutic studies. The aim of this publication is primarily to introduce the KaRhab-registry and to provide a broad overview of the initial data.

## Methods

### Questionnaire

**Fig. 1:**
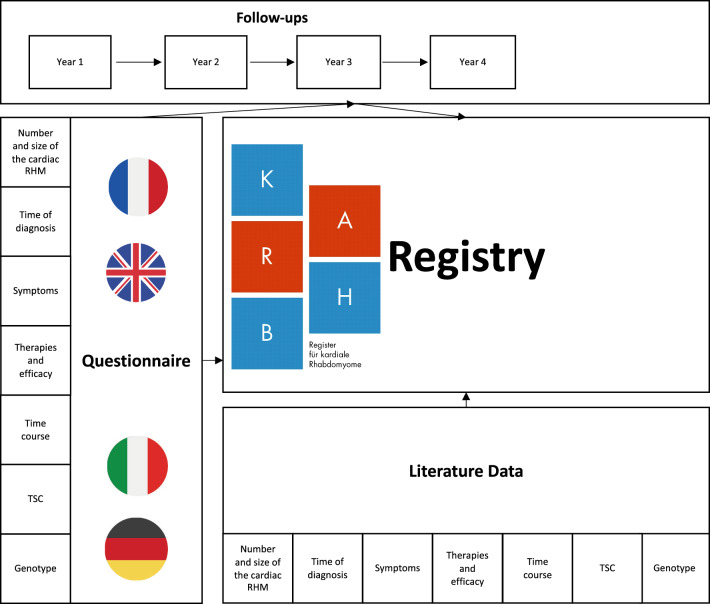
Workflow of the KaRhab Registry

We developed a user-friendly multilingual digital questionnaire for patients with cardiac RHM to collect both clinical and genetic information. The only recruitment criterion is the presence of at least one cardiac RHM. The inquiry is divided into different sections and supposed to cover the topics described in Fig. 1. The questionnaire contains mainly closed questions, which can be answered by selecting several from a list of possible choices (tick boxes). Some questions are open-ended and can be answered in free text. In addition, medical reports can be uploaded for each topic (for example discharge letters, genetic and echocardiography findings). The questionnaire was developed mainly in collaboration between pediatric cardiologists, neuro-pediatricians and human geneticists. The questionnaire is provided via the Research Electronic Data Capture (REDCap) software. Data entry can be interrupted, completed, corrected or deleted at any time by using a case-specific return code. The questionnaire was initially published in German in the year 2021. This was followed a short time later by a version in English, French and Italian. Data collection at the beginning was carried out by specially trained employees of the KaRhab team in Leipzig. Data entry by patients or their relatives on their own behalf is also possible, after which a verification of the entered data is performed by the KaRhab team. A detailed overview of the KaRhab Questionnaire can be found in appendix B.

### Literature Research

To include literature data a systematic PubMed research was performed by using the keywords HEART TUMOR or CARDIAC RHABDOMYOMA. Case reports published from January 2021 to May 2023 were included, in which patients with at least one cardiac RHM were reported. In addition to this, an indication of the number and symptomatology of RHM had to be found. Overlapping cases were excluded.

### Ethics

The study was approved by the ethics committee of the University of Leipzig (No. 187/21-ek). All participants or their legal guardians gave their informed and voluntary consent according to the German data protection standards and the European General Data Protection Regulation. Future participants will be informed in sufficient detail about the general conditions and the data protection concept. The inquiry and further investigations will be performed based on the Declaration of Helsinki. The data protection concept provides for the personal data to be stored on a different server than the pseudonymized health data. Only KaRhab team members can link the health data with the pseudonymized data. This means that retrospective linking of the data, e.g. to delete data when participants wish to withdraw from the registry, remains possible while still ensuring a high standard of data security.

### Data analysis

Data Analysis was conducted in R [17].

## Results

### Participants

Since the launch of the registry in 2021, a total of 14 individuals meeting the inclusion criteria have completed the questionnaire. All patients came from or were once treated in the Heart Center Leipzig or the TSC Center Leipzig. Simultaneously, our literature search yielded case reports and case series encompassing 53 individuals with cardiac rhabdomyomas during the same period. Thus, our analysis relates to a cohort of 67 cases. Among this group, sex was documented in 50 cases, with 22 individuals identified as female (44%) and 28 as male (56%). Age data were specified in 49 cases. Predominantly, the age range did not exceed 30 years, with three exceptions where individuals surpassed this age threshold. Among those younger than 30, the median age was 2.92 years (mean=6.46 years, SD=7.57). Detailed medical and diagnostic reports were only available for the cases included in the registry.

### Diagnosis

Cardiac RHM was diagnosed prenatally in 31 of a total of 54 cases (57.4%) with known time of diagnosis. The time of diagnosis was between 19 and 35 weeks of gestation (median=23 weeks. Mean=26, SD=5.55). In 23 cases cardiac RHM were diagnosed after birth (42.6%). With the exception of 2 cases describing diagnosis after 30 years of age, postnatal diagnosis occurred between the first day of life and 6 months of age. In 64/67 (95.5%) of cases, the cardiac RHM were detectable during ultrasound examination.

### Localization

**Fig. 2:**
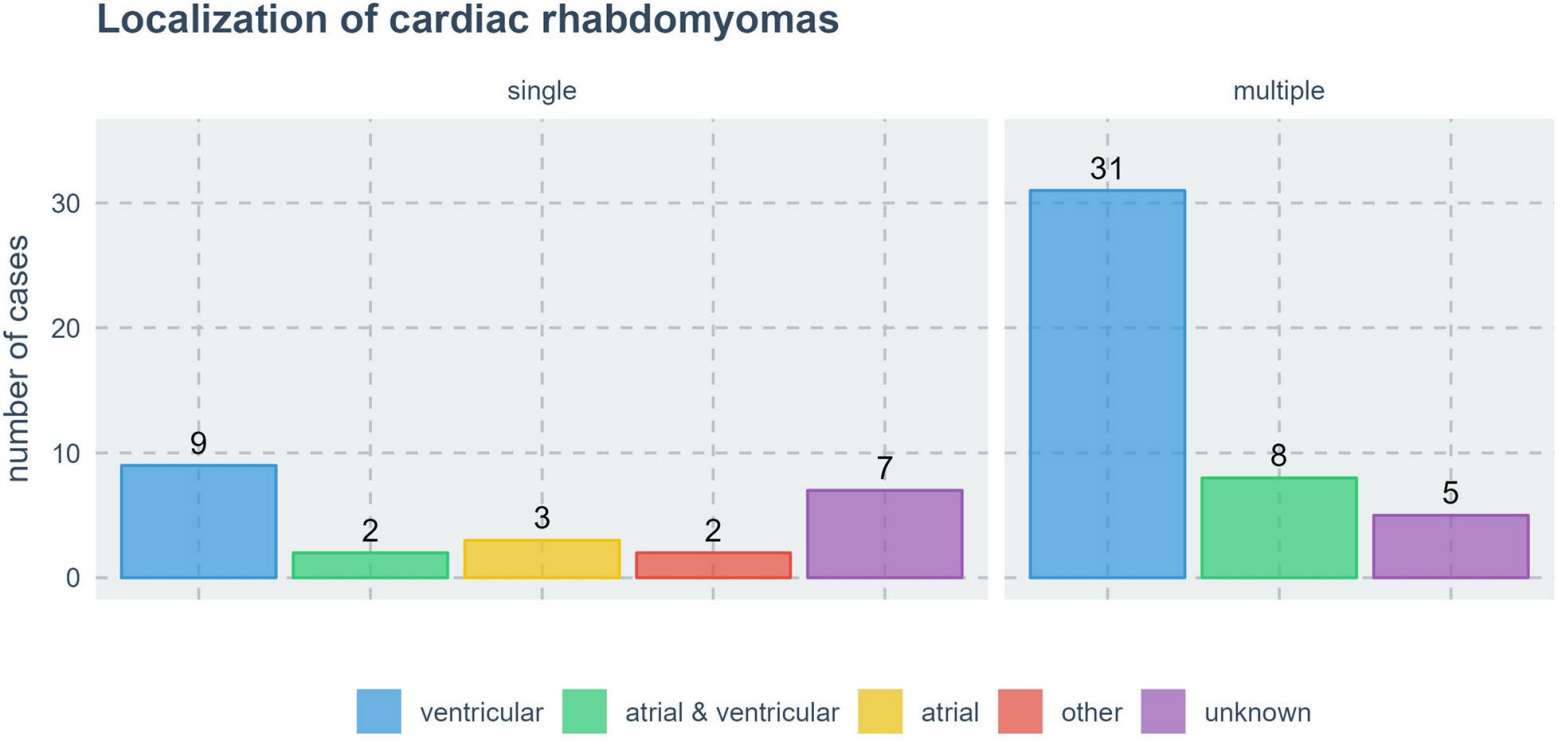
Localization of cardiac RHM. Single: Occurrence of a solitary cardiac RHM. Multiple: Occurrence of more than one cardiac RHM

Out of 67 cases, cardiac RHM presented as a solitary occurrence in 23 cases (34.3%). Among these, 11 cases (47.8%) involved an isolated RHM affecting at least one ventricle. Specifically, the right ventricle was affected in 5 cases, the left ventricle in 3 cases, and both ventricles or more in 3 cases. In 3 out of the 23 cases, a solitary cardiac RHM was exclusively found in the right atrium. In 2 cases out of 23, cardiac RHM occurred in other locations, namely the atrial septum, valves and pericardium. In 7 out of the 23 cases with solitary cardiac RHM, the exact location was unspecified (Fig. 2). Cardiac RHM presented in a multiple pattern in 44 out of 67 cases (65.7%). Location information was available in 39 cases. In all instances, RHM were observed in at least one of the ventricles. Specifically, multiple RHM affecting both ventricles were found in 25 cases. Multiple RHM affecting only the left ventricle were found in 10 cases, RHM affecting only the right ventricle were noted in 4 cases (Fig. 2).

### Symptoms

**Fig. 3:**
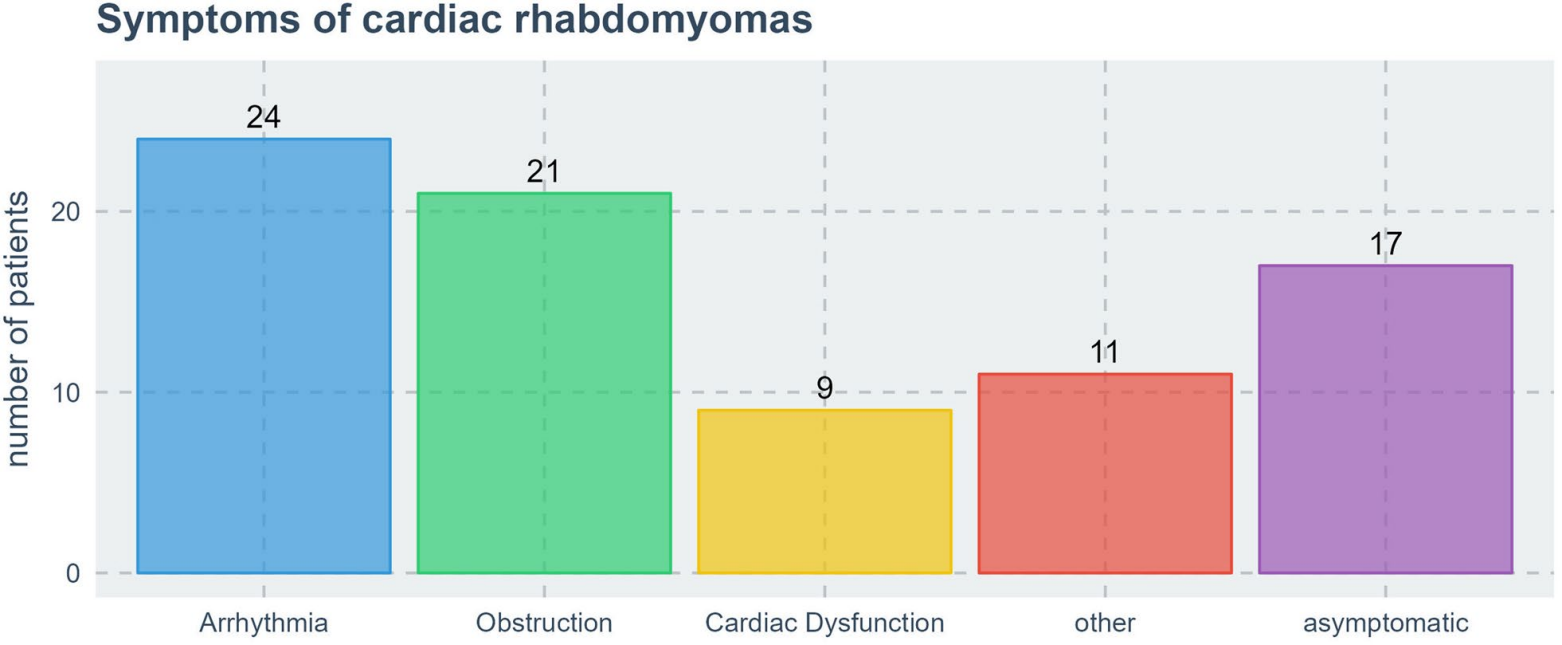
Symptoms caused by cardiac RHM

In 50 of 67 cases (74.6%), cardiac RHM caused symptoms. In the 42 lature cases 31 were seen to be symptomatic (73,8%) whereas 8 of the 14 registry patients were symptomatic (57,1%). Arrhythmia was reported in 24 patients (36.9%), obstruction in 21 patients (32.3%), and cardiac dysfunction in 9 patients (13.8%) Fig. 3. In 11 cases other cardiac symptoms were reported (16.9%) including valvular regurgitation in 5 cases and complete obliteration of cavity in 2 cases (giant RHM) (Fig. 3). Obstruction affected In 17 cases the outflow tract and in 4 cases the inflow tract. Among cardiac arrhythmias, supraventricular tachycardia was reported in 6 cases, bradycardia in 3, ventricular tachycardia in 1 case, and extrasystoles in 1 case. Other singular or unspecified ECG changes were seen in 14 cases.

### Therapies

19 of a total of 43 individuals required intensive care treatment. 37 individuals received pharmacological treatment. Catheter intervention was performed in 3 individuals, and 7 cardiac rhabdomyomas were treated by surgery. Among the 37 pharmacologically treated individuals, 32 received mTOR-inhibitors.

### Association with TSC

**Fig. 4:**
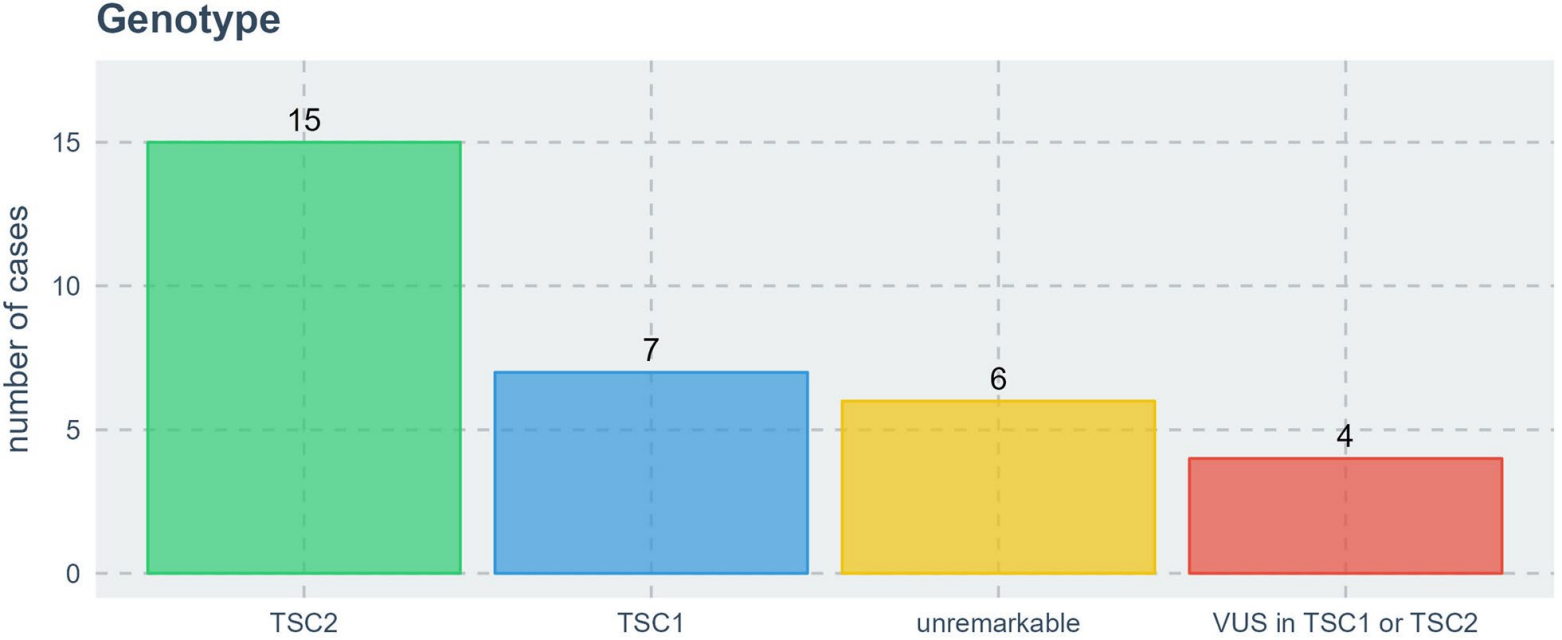
Results of genetic testing in all cases with known performance of genetic testing

51 of 67 individuals were diagnosed with TSC (76.1%), either because they met clinical criteria for TSC and/or had a positive genetic testing. In 4 cases the presence of TSC was unknown. Performance of genetic testing was reported in 32 individuals. Of these, a pathogenic variant was found in *TSC2* in 15 cases (46.9%), a pathogenic variant in *TSC1* in 7 cases (21.9%), an unremarkable result in 6 cases (18.8%), and a Variant of Unknown Significance (VUS) in *TSC1* or *TSC2* in 4 cases (12.5%) (Fig. 4).

## Discussion

For particularly rare diseases, specific treatment guidelines are often missing, and even specialized centers may have limited experience with such cases. This is evident in the example of cardiac RHM. Now that causal therapy in the form of mTOR inhibitors is available, there is an urgent need for a structured overview of treatment strategies, successes, and failures in cardiac RHM. To achieve this, we initiated the KaRhab registry, aiming to accumulate a sufficient number of cases to develop specialized and individualized therapeutic guidelines. To this end, we developed the questionnaire in collaboration with pediatric cardiologists, neuro-pediatricians, and geneticists. This ensures that the collected data will optimally address the most pressing questions regarding cardiac RHM and contribute to the development of therapeutic strategies. For ease of data entry by patients and their relatives and to overcome accessibility barriers, we implemented the questionnaire using the REDCap software. This platform also enables annual re-contacting of the participants to obtain longitudinal data. To ensure a diverse participant pool, we published the questionnaire in different languages, with additional languages planned.

### Overview

While recruitment of new patients continues, this initial overview of our cohort confirms the current understanding of cardiac RHM as a detectable tumor entity using widely available ultrasound. Prenatal diagnosis of cardiac RHM is also not uncommon. A large proportion of cases remain asymptomatic, though some severe cases require treatment. The observed heterogeneity in treatment of cardiac RHM is remarkable. This may be due to the absence of standardized therapy methods and publication bias. Notable differences exist between the literature data and the participants’ registry data, likely because published case studies often focus on severe cases and their treatment attempts. It is mainly singular therapy trials in particularly unusual cases that get published, whereas asymptomatic cases are rarely documented. This bias should be considered in the more detailed evaluation planned for the future.

### Limitations

The current number of cases is still too low for a comprehensive investigation, particularly concerning more specific questions about symptoms, therapy, and genetic variants. To further increase the number of cases, we conducted a systematic literature search to add previously published cases to the registry. This revealed the following limitations: unlike the registry data, longitudinal data cannot be obtained from literature sources. Moreover, literature data lacks specificity to the questionnaire, thereby limiting its ability to address all our questions. For certain items in our questionnaire, there is an unusual amount of missing data. For example, genetic data is often not shared, likely because the published cases are described from a cardiology or cardiac surgery perspective. While acknowledging these limitations, we firmly believe that a high caseload is of major importance.

### Future steps

The first phase of patient recruitment highlighted the difficulties and time-consuming nature of setting up a registry for a rare condition like cardiac RHM. This task becomes even more challenging when aiming to develop sufficient data from the registry as the basis for a treatment regimen. Our experience shows that creating a registry of sufficient size requires high visibility and cross-center professional collaboration. To achieve a significant increase in the number of cases, we plan to enhance the visibility of the registry on an international level. We aim to establish international collaborations and directly involve patient associations. A detailed evaluation is planned for the future once the number of cases exceeds 100, patients from different centers are included, and longitudinal data are obtained.

## Conclusion

The heterogeneity in therapeutic strategies highlights the lack of evidence-based experience in optimally treating cardiac RHM. Considering the positive effects of mTOR inhibitors in published cases, further research is essential. Our registry study aims to better characterize cardiac RHM and standardize therapies so that more patients can be diagnosed earlier and treated sooner. Thus, we eagerly anticipate the participation of individuals from all over the world.

## Supplementary Information

Supplementary file 1.

Supplementary file 2.

## Data Availability

The datasets used and/or analyzed during the current study are enclosed in appendix A. The R code is available from the corresponding author on reasonable request.

## List of abbreviations

RHM: Rhabdomyoma
TSC: Tuberous Sclerosis Complex
mTOR: Mammalian target of rapamycin
VUS: Variant of Unknown Significance
